# Unraveling the Local Relation Between Tissue Composition and Human Brain Mechanics Through Machine Learning

**DOI:** 10.3389/fbioe.2021.704738

**Published:** 2021-08-17

**Authors:** Kevin Linka, Nina Reiter, Jasmin Würges, Martin Schicht, Lars Bräuer, Christian J. Cyron, Friedrich Paulsen, Silvia Budday

**Affiliations:** ^1^ Institute of Continuum and Material Mechanics, Hamburg University of Technology, Hamburg, Germany; ^2^ Institute of Applied Mechanics, Department Mechanical Engineering, Friedrich-Alexander-University Erlangen-Nürnberg, Erlangen, Germany; ^3^ Institute of Functional and Clinical Anatomy, Faculty of Medicine, Friedrich-Alexander-University Erlangen-Nürnberg, Erlangen, Germany; ^4^ Institute of Material Systems Modeling, Helmholtz-Zentrum Hereon, Geesthacht, Germany; ^5^ Department of Operative Surgery and Topographic Anatomy, Sechenov University, Moscow, Russia

**Keywords:** human brain, viscoelasticity, constitutive modeling, microstructure, mechanical properties, artificial neural network, extracellular matrix

## Abstract

The regional mechanical properties of brain tissue are not only key in the context of brain injury and its vulnerability towards mechanical loads, but also affect the behavior and functionality of brain cells. Due to the extremely soft nature of brain tissue, its mechanical characterization is challenging. The response to loading depends on length and time scales and is characterized by nonlinearity, compression-tension asymmetry, conditioning, and stress relaxation. In addition, the regional heterogeneity–both in mechanics and microstructure–complicates the comprehensive understanding of local tissue properties and its relation to the underlying microstructure. Here, we combine large-strain biomechanical tests with enzyme-linked immunosorbent assays (ELISA) and develop an extended type of constitutive artificial neural networks (CANNs) that can account for viscoelastic effects. We show that our viscoelastic constitutive artificial neural network is able to describe the tissue response in different brain regions and quantify the relevance of different cellular and extracellular components for time-independent (nonlinearity, compression-tension-asymmetry) and time-dependent (hysteresis, conditioning, stress relaxation) tissue mechanics, respectively. Our results suggest that the content of the extracellular matrix protein fibronectin is highly relevant for both the quasi-elastic behavior and viscoelastic effects of brain tissue. While the quasi-elastic response seems to be largely controlled by extracellular matrix proteins from the basement membrane, cellular components have a higher relevance for the viscoelastic response. Our findings advance our understanding of microstructure - mechanics relations in human brain tissue and are valuable to further advance predictive material models for finite element simulations or to design biomaterials for tissue engineering and 3D printing applications.

## 1 Introduction

The human brain is a fascinating organ, which has been studied intensively by researchers from various fields but still remains incompletely understood. Recent studies have highlighted the important role of mechanical properties and forces for certain processes during brain development (Budday et al., 2015b; Koser et al., 2016; Thompson et al., 2019; injury Meaney et al., 2014; Hemphill et al., 2015; Keating and Cullen, 2021), and disease (Murphy et al., 2016; Barnes et al., 2017; Gerischer et al., 2018; Park et al., 2018). Mechanical instabilities seem to underlie cortical folding during brain development (Budday et al., 2015b; Garcia et al., 2018), and brain cells react to their mechanical environment by converting mechanical stimuli into neural signals through mechanotransduction, which again triggers cellular or extracellular reactions (Moshayedi et al., 2010, 2014; Tyler, 2012; Franze et al., 2013; Blumenthal et al., 2014; Humphrey et al., 2014; Irianto et al., 2016; Koser et al., 2016; Urbanski et al., 2016; Barriga et al., 2018). Consequently, the human brain continuously changes its microstructure, mechanical properties, and shape during its lifetime (Budday and Kuhl, 2020), which makes it one of the most complex organs in the human body. For many pathological conditions, such as degenerative diseases, microstructural changes have been investigated by neuropathologists (Alafuzoff, 2018). However, the link between changes in microstructural components, the corresponding tissue mechanics, and the effect induced through the mechanosensing of cells remains to be clarified (Begonia et al., 2010). Better understanding whether and how microstructural components contribute to the macroscopic mechanical behavior of brain tissue is key to gain further insights into the mechanisms underlying mechanics-related injury and disease. In addition, computational models based on nonlinear continuum mechanics can be a valuable tool to predictively understand the processes in the human brain (Goriely et al., 2015; Budday et al., 2020a). Eventually, they could even be used to assist diagnosis and treatment of neurological disorders or the detailed planning of surgical procedures (Weickenmeier et al., 2017a; Zarzor et al., 2021). In this respect, understanding the link between microstructure and mechanics of brain tissue can help to develop more realistic material models that capture local variations in tissue properties (Budday et al., 2020b; Reiter et al., 2021).

**FIGURE 1:**
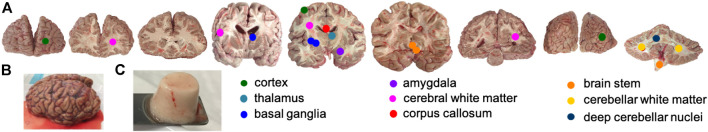
Human brain tissue samples. **(A)** Locations where **(C)** specimens were harvested from **(B)** complete brains. Circles marking the location of specimen extraction were enlarged for better visibility.

**FIGURE 2:**
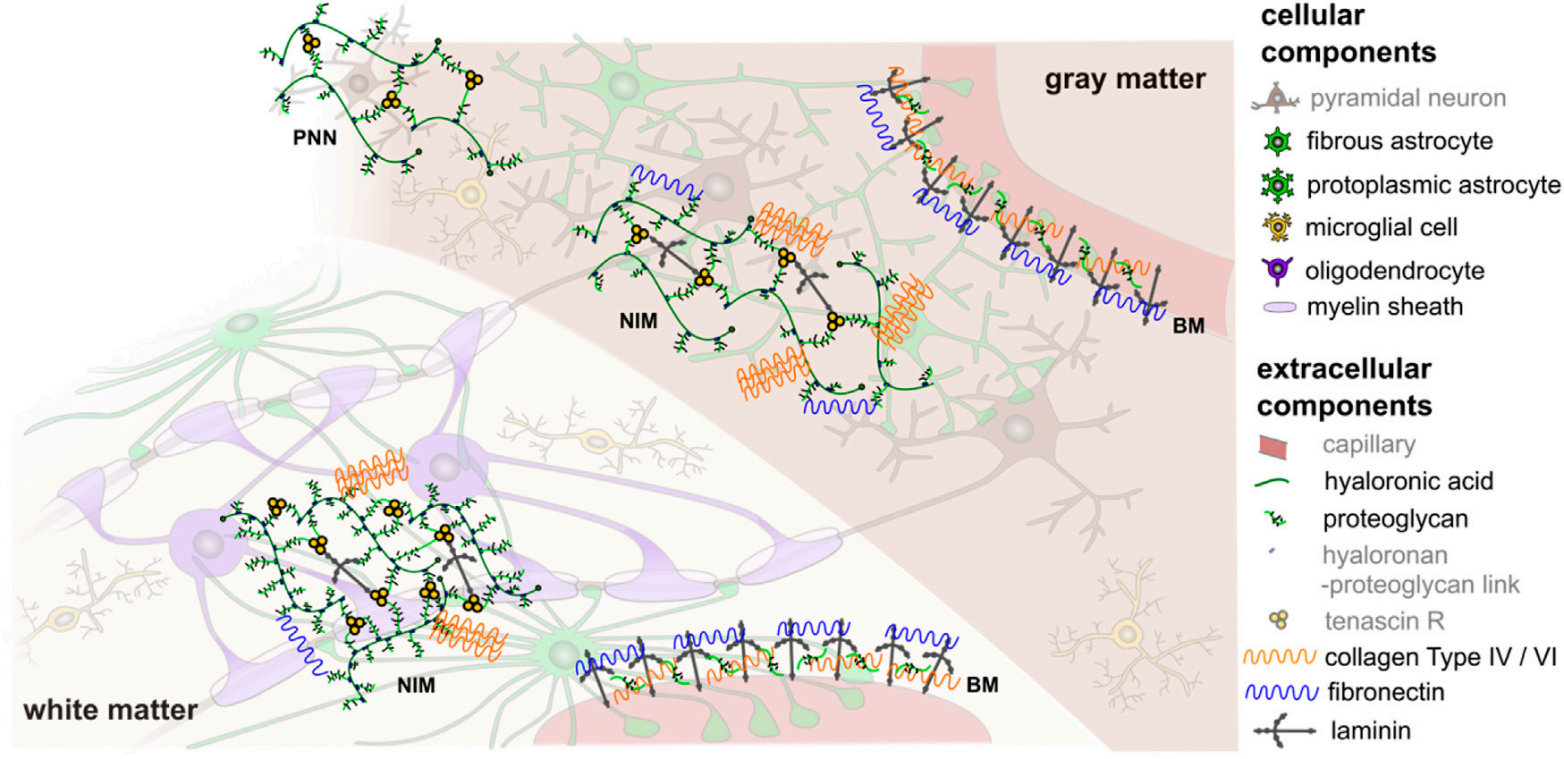
Schematic illustration of the cellular and extracellular components of human brain tissue (components greyed out are not further considered in the present work). White matter contains oligodendrocytes, which wrap myelin sheath around axons, as well as fibrous astrocytes and microglia. Gray matter contains mainly neurons, protoplasmic astrocytes, and microglia. The extracellular matrix has three principal compartments: the basement membrane (BM), which lines cerebral microvessels and the pial surface, the neural interstitial matrix (NIM), which is diffusely distributed in the brain’s interstitial space, and perineuronal nets (PNN), which surround inhibitory interneurons in certain areas of gray matter. In different compositions, these compartments contain proteoglycans, hyaluronic acid, link proteins, glycoproteins (e.g., tenascin, laminin, fibronectin), and non-fibrillar collagens type IV and VI (Lau et al., 2013). This is a schematic figure for identification purposes only with no claim of being complete or true to scale. Reprinted from Budday et al. (2020b) with permission from Elsevier.

One challenge when modeling the behavior of brain tissue is the exceptional heterogeneity in mechanical properties resulting from regional differences in the microstructure due to local functional demands. While we can clearly distinguish two tissue types on the macroscopic scale, gray and white matter (see Figure 1), the microstructure will locally vary significantly–even within those regions. Previous research on the microstructural composition of brain tissue has largely focused on the brain’s cellular components with an emphasis on neurons. However, also support cells called neuroglia as well as the extracellular matrix highly contribute to normal and abnormal brain functioning (Lau et al., 2013). In general, the neuroglia can be divided into macroglia and microglia. The macroglia originate from the neural tube, i.e., are of ectodermal origin, the microglia originate from the mesoderm. The most important types of macroglia are astrocytes with mechanical and metabolic tasks such as maintaining the blood-brain barrier, oligodendrocytes, which support transduction through myelin sheath formation, and ependymal cells, which line the inner cerebrospinal fluid spaces (the latter, however, do not play a role for the investigations made in this work). The microglial cells are the macrophages of the central nervous system. All the glial cells mentioned (except ependym) have numerous cell processes. Unlike nerve cells, glial cells can proliferate. They support neurons and contribute to tissue homeostasis, and thereby influence the mechanical properties of the tissue. The majority of brain tumors originate from glial cells, which further highlights their importance for pathological processes. Furthermore, extracellular matrix components, such as proteoglycans, hyaluronic acid, and non-fibrillar collagens surround the cells, as illustrated in Figure 2 (Novak and Kaye, 2000; Lau et al., 2013; Budday et al., 2020b). They embody approximately 40% of the brain’s volume during development (Rauch, 2004) and 20% during adulthood (Bellail et al., 2004; Rauch, 2004; Oohashi et al., 2015) and might thus also play an important role in brain tissue mechanics.

An important challenge associated with the aim to define the relations between mechanics and microstructure is to reliably and consistently quantify these features. In terms of mechanics, the exceptionally complex mechanical response–characterized by nonlinearity, compression-tension-asymmetry, conditioning effects, and stress relaxation–makes it impossible to describe the mechanical properties through a single stiffness value. The measured modulus highly depends on the loading mode, strain regime, strain rate, drainage conditions, and length scale (Chatelin et al., 2010; Budday et al., 2020a). Therefore, to account for nonlinear and time-dependent effects, it is important to, on the one hand, perform large-strain biomechanical tests combining cyclic and stress relaxation experiments, and, on the other hand, analyze the corresponding experimental data based on the theory of nonlinear continuum mechanics (Miller and Chinzei, 1997; Bilston et al., 2001; Miller and Chinzei, 2002; Prevost et al., 2011; Rashid et al., 2012; Budday et al., 2017a, 2020a).

In terms of microstructure, previously used techniques to investigate tissue components include histological and immunohistochemical stains or western blots (Yang and Mahmood, 2012; Alafuzoff, 2018), which can provide information about the presence, morphology, local distribution, or molecular weight of certain tissue components. In neuropathology, they are frequently used to distinguish the diseased from the healthy state. Yet, these methods only show a small section of the tissue and fail to provide trustworthy quantitative values on the amount of specific molecules (Taylor and Levenson, 2006; Yang and Mahmood, 2012; Dabbs, 2014). An alternative enabling a more reliable quantitative assessment is another immunological method called enzyme-linked immunosorbent assay (ELISA). It is an extremely sensitive colorimetric method to quantify biological molecules by using antibody-antigen complexes (Gan and Patel, 2013). As ELISA is an accurate, cost-effective, and quick technique, it has become a widely used method for the qualitative or quantitative analysis of molecules in versatile fields. Still, it has to the best of the authors’ knowledge not been used in the context of microstructure - mechanics relations in brain tissue yet.

Previous studies relating microstructure and mechanics of brain tissue have indicated that tissue stiffness increases with myelination during development in white matter (Weickenmeier et al., 2016; Weickenmeier et al., 2017b), negatively correlates with the fractional anisotropy (a structural parameter from magnetic resonance imaging and diffusion tensor imaging) (Budday et al., 2017a), and negatively correlates with the density of cell nuclei (Antonovaite et al., 2018; Budday et al., 2020b). However, these studies were based on the evaluation of imaging data which quantify the tissue composition much less accurately than ELISAs. In addition, they evaluated only the correlation between composition and individual mechanical parameters, such as the shear modulus, nonlinearity, or stress relaxation, but did not consider the entire loading history.

While first studies have successfully incorporated distinct microstructural parameters into analytic constitutive laws for brain tissue (Budday et al., 2020b: Reiter et al., 2021), data-driven approaches such as machine learning bear the potential to open up a much more comprehensive view. First attempts to use machine learning for relating tissue microstructure to macroscopic mechanical properties used simple end-to-end model architectures (Liang et al., 2017). To overcome the large amount of data required by such approaches, (Linka et al., 2021) recently introduced constitutive artificial neural networks (CANNs) as a novel machine learning architecture that incorporates substantial prior knowledge from materials theory. Thereby, it can learn to describe and in fact also predict the nonlinear behavior of soft biological tissue from information about its microstructure and composition based on a much smaller amount of training data than previous methods.

In this paper, we generalize the concept of CANNs to viscoelasticity and apply it to experimental data from human brain tissue. These data include results from large-strain mechanical tests and compositional analysis using ELISAs. Using relevance propagation, a concept of explainable artificial intelligence (Samek et al., 2021), we identify the importance of the different tissue constituents for the mechanical response of human brain tissue, where quasi-elastic and viscous effects show distinct regional trends.

## 2 Materials and Methods

### 2.1 Human Brain Tissue

We obtained five whole human brains including the cerebrum, cerebellum, and brainstem (see Figure 1B) from one female and four male body donors who had given their written consent to donate their body to research. The body donors were aged between 62 and 92 years and none of them had suffered from any neurological disease known to affect the microstructure of the brain (see Table 1). We note that for subjects 3 and 5, we could not find metastases in the brain. The brains 1-3 and 5 were immersed in cerebrospinal fluid surrogate (CSFS) during transport. Brain 4 was kept in phosphate buffered saline solution (PBS). We received the brains between 9 and 24 h *post mortem* and directly cut them into 1 cm thick coronal slices. After that, we kept the slices refrigerated at 4 °C in CSFS or PBS until mechanical testing. We completed the mechanical experiments within 72 h *post mortem*. The study was approved by the Ethics Committee of Friedrich-Alexander University Erlangen-Nürnberg, Germany, with the approval number 405_18 B.

**TABLE 1:** Human brains.

Brain	Sex	Age	Cause of death
1	Male	92	dotage
2	Female	62	liver and kidney failure
3	Male	68	metastasizing bronchial carcinoma
4	Male	75	cardiac insufficiency
5	Male	75	metastasizing bronchial carcinoma

#### 2.1.1 Specimen Preparation

The samples for the ELISAs of brain 3–5 were extracted directly after cutting the brains into slices to minimize the *post mortem* degradation of proteins before the samples were frozen and stored at −20°C. Figure 1A shows the anatomical brain regions that we included in our study. For brains 1 and 2, we extracted the ELISA samples simultaneously with the respective mechanical sample. Therefore, the ELISA samples of those two brains were frozen at different *post mortem* times.

The specimens for the mechanical characterization were extracted directly next to the locations of the ELISA specimens and were prepared right before testing. We used a biopsy punch to extract cylindrical samples of 8 mm diameter, as shown in Figure 1C. We punched the specimens out of the coronal slices while they were immersed in CSFS so that the cylindrical specimens could slide out of the biopsy punch without adhering to it. Like this, we could ensure that our samples only experienced small deformations before they were probed mechanically. If the small cylinders had a height of more than 6 mm, we carefully shortened them with a surgical scalpel. The specimen height ranged between 3.5 and 6 mm. For most regions, it was possible to extract homogeneous specimens of this size. The only exception were the deep cerebellar nuclei: The corresponding samples contained a certain amount of cerebellar white matter, which might affect the results.

We included a total number of *n* = 86 samples for mechanical experiments and *n* = 78 samples for the ELISAs, as, for eight of the ELISA samples, we were able to extract two corresponding mechanical specimens. Table 2 summarizes the samples extracted from each brain region.

**TABLE 2:** Samples for mechanical testing and ELISA.

Anatomical region		Number of mechanical samples	Number of ELISA samples
Cortex	C	15	15
Thalamus	TH	4	4
Basal ganglia	BG	14	14
Amygdala	AMY	3	3
Corona radiata	CR	19	19
Corpus callosum	CC	10	5
Brainstem	BS	15	12
Cerebellar white matter	cWM	5	5
Deep cerebellar nuclei	cNC	1	1

#### 2.1.2 Mechanical Testing

**FIGURE 3:**
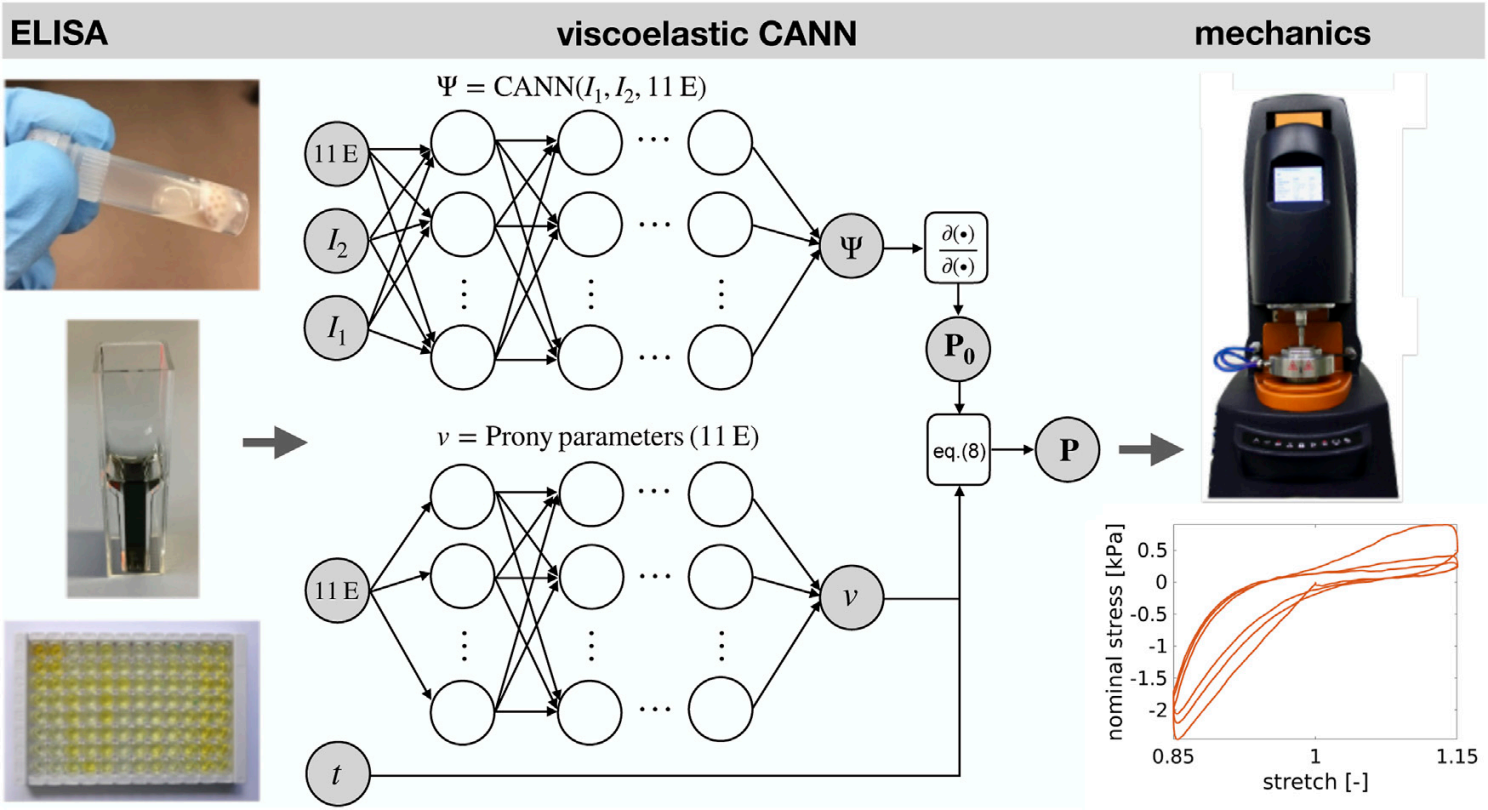
11 ELISA parameters (11 E) and strain data from mechanical tests (in the form of invariants *I*_1_, *I*_2_,… ) form the input of an extended type of constitutive artificial neural network. Machine learning adjusts its stress output to the one measured experimentally. Thereby, the neural network learns to describe the mechanical behavior of brain tissue and to predict it from the ELISA parameters. The extended CANN consists of a standard CANN block and a parallel deep neural network computing the Prony parameters of the viscoelastic constitutive behavior. Note that the stress response **P** is computed by a recursive update in time *t*. The hidden neurons in the network are illustrated as blank circles, while the associated network weights are depicted as black arrows.

We used a Discovery HR-3 rheometer from TA instruments (New Castle, Delaware, United States) to measure the tissue response under compression and tension (see Figure 3B). After calibration, we fixed the specimens to the upper and lower specimen holder using sandpaper and superglue. We waited 30–60 s to let the glue dry before immersing the specimen in PBS to keep it hydrated during the experiment. We conducted all tests at 37°C. We first applied three cycles of compression and tension with a loading velocity of 40 *μ*m/s and minimum and maximum stretches of *λ* = [*H* + Δ*z*]/*H* = 0.85 and *λ* = 1.15, where *H* denotes the initial specimen height and Δ*z* the displacement in the direction of loading. Subsequently, we performed a compression relaxation test at *λ* = 0.85 with a loading velocity of 100 *μ*m/s and a holding period of 300 s, and a tension relaxation test at *λ* = 1.15, with the same loading velocity and holding period. We recorded the corresponding force *f*_*z*_ and determined the nominal stress as *P*_exp_ = *f*_*z*_/*A*, where *A* is the cross-sectional area of the specimen in the undeformed configuration.

#### 2.1.3 ELISA

We used commercially available enzyme-linked immunosorbent assay kits (ELISAs from Cloud-Clone and Cusabio, Wuhan, China) to quantify the amount of GFAP, MBP, Iba1, Col I, Col IV, CS, LAM, FN, HA, Col VI, and LUM (see Table 3) in samples of protein extracts from human brain tissue (see Figure 3B). We isolated protein out of the brain samples using 300 *μ*l Triton buffer containing 0.2% protease and 0.2% phosphatase inhibitors. The brain solutions were incubated on ice for 30 min. After centrifuging at 13,000 rpm and 4°C for 5 min, we diluted the solutions to 1 ml with Triton buffer to ensure that we could perform all ELISAs. Subsequently, we decanted the supernatant and measured the protein concentration with a Bradford assay. The analysis was performed using a microplate spectrophotometer (ELISA-reader) at a wavelength of 450 and 405 nm for measuring the absorbance. The received optical density results for the standard dilutions were then utilized to create standard curves using the software MARS Data Analysis from BMG Labtech and the 4- or 5-parameter best fit. By comparing with the standard series and the determined values for antigen concentration (protein concentration), we calculated the content of the protein in ng/total protein in mg in each sample.

**TABLE 3:** Proteins investigated by ELISA.

Investigated protein		Manufacturer	Cat. nr	ELISA type	Detection range [ng/ml]
Glial fibrillary acidic protein	GFAP	Cloud-Clone	SEA068Hu	sandwich	0.156–10
Ionized calcium-binding adapter molecule 1	Iba1	Cloud-Clone	SEC288Hu	sandwich	0.0312–2
Myelin basic protein	MBP	Cloud-Clone	SEA539Hu	sandwich	0.156–10
Hyaluronic acid	HA	Cusabio	CSB-E04805h	sandwich	0.156–10
Chondroitin sulfate	CS	Cloud-Clone	CEA723Ge	competitive	0.03906–10
Lumican	LUM	Cloud-Clone	SEB496Hu	sandwich	0.312–20
Collagen I	Col I	Cloud-Clone	SEA571Hu	sandwich	0.156–10
Collagen IV	Col IV	Cloud-Clone	SEA180Hu	sandwich	7.8–500
Collagen VI	Col VI	Cloud-Clone	SED123Hu	sandwich	0.78–50
Fibronectin	FN	Cloud-Clone	SEA037Hu	sandwich	1.56–100
Laminin	LA	Cloud-Clone	SEA082Hu	sandwich	7.8–500

### 2.2 Viscoelastic Constitutive Artificial Neural Network

Constitutive artificial neural networks (CANNs) have recently been introduced as a novel machine learning architecture and shown to be a powerful tool for using machine learning for mechanical constitutive modeling (Linka et al., 2021). To empower them to deal with brain tissue, we use herein an extension that combines a standard CANN for the quasi-elastic response to loading (i.e., on very short time scales) with an additional, parallel deep neural network computing so-called Prony parameters accounting for the time-dependent stress relaxation observed in viscoelastic materials. In the following, we discuss the technical details of this architecture under the assumption that brain tissue can be modeled as a quasi-linear viscoelastic incompressible isotropic material.

#### 2.2.1 Constitutive Artificial Neural Networks

To reduce the amount of training data required to learn the mechanical constitutive behavior of materials, CANNs exploit the role of symmetries in materials theory. For the simple special case of incompressible isotropic materials, on which we focus herein, this means that CANNs capture the constitutive behavior of materials via a deep neural network mapping the first (*I*_1_) and second (*I*_2_) principal invariants

$$I_{1}=tr\left(C\right),\;I_{2}=\frac{1}{2}\left[{\left(trC\right)}^{2}-trC^{2}\right],$$ (1)

of the right Cauchy-Green deformation tensor **C** = **F** ^T^ **F** on the strain energy

$$\Psi \left(C\right)=\Psi \left(I_{1},\;I_{2}\right).$$ (2)

Here, **F** denotes the deformation gradient. The first and second Piola-Kirchhoff stress tensors **P**_0_ and **S**_0_ can be obtained in this setting simply by symbol-to-symbol automatic differentiation of the output Ψ of the neural network. We note that the first Piola-Kirchhoff stress tensor **P**_0_ corresponds to the nominal stress recorded during the experiments described in section 2.1.2. An important feature of CANNs is that their input is formed not only by invariants of the deformation state but also by any kind of additional quantities potentially carrying information about the constitutive behavior of the material of interest. In our case, these additional quantities are the 11 parameters measured in our experiments by ELISAs (see Figure 3 and section 2.1.3). This architecture enables CANNs to learn not only how to resemble stress-strain curves of brain tissue but also to predict such curves from ELISAs, as discussed in more detail in Linka et al. (2021).

#### 2.2.2 History Dependence

The response to loading of viscoleastic materials is in general governed not only by the current loading but also by the loading history. Skipping herein the complex theory of general nonlinear viscoelastic materials, we adopt the theory of quasi-linear viscoelasticity as introduced by Y. C. Fung in particular for biological tissues (Fung, 2013). This theory allows stress to depend nonlinearly both on strain and time. However, it assumes that the role of strain and time can be separated by a multiplicative split. While this limits the generality of the theory, it has been found that many biological materials of interest can be modeled at least in very good approximation as quasi-linear viscoelastic materials. Within this setting, the history-dependent stress can be expressed by the convolution integral

$$P=\overset{t}{\underset{0}{\int }}g\left(t-s\right)\;\frac{\partial P_{0}}{\partial s}\;ds,$$ (3)

where **P**_0_ denotes the quasi-elastic stress response (i.e., on very short time scales) of a material under a Heaviside strain as approximated by a CANN, and *g* is a kernel function characterizing stress relaxation over time *t*. In this work, we assume a Prony-type kernel function (Taylor et al., 2009)

$$g\left(t\right)=g_{0}+\overset{p}{\underset{i=1}{\sum }}\;g_{i}\;\exp\left[-\frac{t}{\tau_{i}}\right]$$ (4)

with scalar weighting coefficients *g*_*i*_ with a partition of unity property

$$g_{0}+\overset{p}{\underset{i=1}{\sum }}g_{i}=1$$ (5)

and relaxation time constants *τ*_*i*_. The set of Prony parameters is denoted herein by

$$v=\left\{g_{0},g_{1},\tau_{1},g_{2},\tau_{2},\ldots ,\right\}.$$ (6)

The stress response **P** can be split into a long-term elastic and a transient viscoelastic contribution as

$$P=g_{0}\;P_{0}+\underset{h_{i}}{\underset{︸}{\overset{p}{\underset{i=1}{\sum }}\;\overset{t}{\underset{0}{\int }}\;g_{i}\;\exp\left[-\frac{t}{\tau_{i}}\right]\frac{dP_{0}}{ds}\;ds}}$$ (7)

with the i-th history integral *h*_*i*_(*t*). Following Goh et al. (2004), this formula can be used to evaluate the current stress **P** over time *t* efficiently in a time-discrete setting with time points *t* ^*n*^ by the pair of recursive formulae

$$\begin{array}{c} P\left(t^{n+1}\right)=g_{0}P_{0}\left(t^{n+1}\right)+\overset{p}{\underset{i=1}{\sum }}h_{i}\left(t^{n+1}\right),\\ h_{i}\left(t^{n+1}\right)=\exp\left(-\Delta t/\tau_{i}\right)h_{i}\left(t^{n}\right)+g_{i}\frac{1-\exp\left(-\Delta t/\tau_{i}\right)}{\Delta t/\tau_{i}}\left[P_{0}\left(t^{n+1}\right)-P_{0}\left(t^{n}\right)\right]. \end{array}$$ (8)

Altogether, the extended type of CANN used herein computes the quasi-elastic stress response **P**_0_ of the materials and its Prony parameters by two separate, parallel deep neural networks. Subsequently, it computes the time-dependent current stress using Eq. (8), as illustrated in Figure 3. Note that the dependence of the stress response on the 11 ELISA values is learned by the viscoelastic CANN. In agreement with previous studies (Prange and Margulies, 2002; Budday et al., 2015a; Budday et al., 2017b), we used *p* = 2 Prony terms in our machine learning architecture.

#### 2.2.3 Model Training and Hyperparameter Tuning

To train our viscoelastic CANNs, we used Adam optimization Kinga and Adam, (2015) for minimizing the mean-squared-error (MSE) loss function

$$MSE=\underset{i}{\sum }|P_{zz}^{i}-P_{\text{exp}}^{i}{|}^{2}.$$ (9)

Here, $P_{zz}^{i}$ is the stress component in loading direction as computed by our viscoelastic CANN and $P_{\text{exp}}^{i}$ is the corresponding experimentally observed value. The index *i* loops through all experimentally collected stretch-stress tuples included in the training process. Our whole framework was implemented using Keras with TensorFlow backend (Chollet, 2015; Abadi et al., 2020). We used Glorot weight initialization (Glorot and Bengio, 2010) at the beginning of the training and fixed the learning rate at 0.001 during the training of different layers. Training was performed with 250 data pairs in each iteration (also referred to as batch size), which was chosen corresponding to the amount of cyclic stress-stretch data points of a single tissue specimen. Before starting the actual training, we performed a hyperparameter tuning for the network topology, dropout rate, L2-regularization and the activation functions using a Bayesian optimization with a Gaussian process model (Mockus, 1994; Chollet, 2015). This tuning was performed on cyclic loading data of one representative tissue specimen. It led to a CANN architecture with three hidden layers with (32, 32, 48) computational units (neurons) with hyperbolic tangent activation functions, an elu activation function (Clevert et al., 2015) for the output, and a dropout layer after the first hidden layer with a rate of 0.5. For the network computing the Prony parameters, hyperparameter tuning resulted in a single hidden layer with 12 computational units and a sigmoid activation function.

Leave-one-out cross validation (LOO-CV) was used to train the model on the full range of available cyclic loading data. Here, the number of evaluation folds is equal to the number of samples in the data set (*N* = 86). Accordingly, each model was first trained based on *N* − 1 data sets, and then the trained model was applied to the single left out validation sample to evaluate the ability of the trained neural network to generalize (predict). In this way, each individual sample was used for one particular model training as validation sample. Each model instance was trained for 4,000 epochs, and the epoch with the best validation set accuracy was chosen for evaluation purposes to prevent overfitting that might occur after too many epochs. More details on training and validation are provided in the supplementary materials *Model Training and Validation*.

For validation of our trained machine-learning model, we computed for the validation sample the coefficient of determination

$$R^{2}=1-\left(S_{\text{res}}/S_{\text{tot}}\right),\;with\;S_{\text{res}}=\underset{i}{\sum }{\left(P_{\text{exp}}^{i}-P_{11}^{i}\right)}^{2},\;S_{\text{tot}}=\underset{i}{\sum }{\left(P_{\exp^{i}}-{\bar{P}}_{\exp}\right)}^{2},$$ (10)

where ${\bar{P}}_{\exp}$ is the mean of the experimental data points.

#### 2.2.4 Layer-wise Relevance Propagation

A primary goal of this work is the evaluation of the impact of the different compositional parameters measured by the ELISAs on the mechanical properties. To this end, we use the concept of layer-wise relevance propagation. It is a method from the research area of explainable artificial intelligence and particularly suitable for deep neural networks, which map some input through a series of layers to an output layer. The *l*th layer consists of computational units (neurons) passing the values $x_{i}^{l}$ to the next layer, where *i* is the index of the neuron within layer *l*. The propagation of values from layer *l* − 1 to layer *l* can in general be described by

$$z_{ij}^{l}=w_{ij}^{l}x_{i}^{l-1},\;z_{j}^{l}=\underset{i}{\sum }z_{ij}^{l}+b_{j}^{l},\;x_{j}^{l}=n_{j}^{l}\left(z_{j}^{l}\right).$$ (11)

Here $w_{ij}^{l}$ is the weight connecting the neuron *i* in layer *l* − 1 with neuron *j* in layer *l*; $z_{j}^{l}$ is the input neuron *j* in layer *l* receives from all neurons of the previous layer plus the bias $b_{j}^{l}$ of this neuron; $x_{j}^{l}$ is the output this neuron passes to the neurons on the subsequent layer after application of its in general nonlinear activation function $n_{j}^{l}$.

**FIGURE 4:**
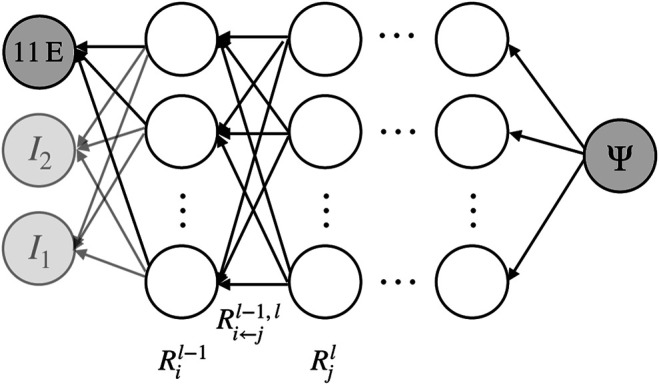
Illustration of layer-wise (backwards) relevance propagation in a CANN. In a trained neural network, recursive application of this scheme from the output layer to the input layer can quantify the relevance of the individual input parameters such as the ELISA values for the output. Neurons are illustrated as empty circles, weights as black arrows.

Within this general setting, layer-wise relevance propagation aims at tracing back a given output to individual components of the input layer of the deep neural network. To this end, it starts at the output layer. Then it recursively computes the relevance score $R_{i}^{l-1}$ of all neurons *i* in layer *l* − 1 from known relevance scores $R_{j}^{l}$ of the neurons *j* in layer *l*, see also Figure 4. In this procedure, the relevance $R_{j}^{l}$ is propagated backwards from layer *l* to layer *l* − 1 by dividing it into relevance contributions $R_{i\leftarrow j}^{l-1,\;l}$ for each neuron *i* in layer *l* − 1, observing the conservation property

$$\underset{i}{\sum }R_{i\leftarrow j}^{l-1,\;l}=R_{j}^{l}.$$ (12)

The relevance of neuron *i* in layer *l* − 1 is then generally computed as

$$R_{i}^{l-1}=\underset{j}{\sum }R_{i\leftarrow j}^{l-1,\;l}.$$ (13)

The key of such relevance propagation schemes is the formula by which the relevance contributions $R_{i\leftarrow j}^{l-1,\;l}$ propagated from layer *l* back to layer *l* − 1 are computed. Herein, we follow Bach et al. (2015) and define

$$R_{i\leftarrow j}^{l-1,\;l}=R_{j}^{l}\;\left[\alpha \frac{z_{ij}^{l+}}{z_{j}^{l+}}+\beta \frac{z_{ij}^{l-}}{z_{j}^{l-}}\right]$$ (14)

where the superscripts − and + denote the negative and positive parts of $z_{ij}^{l}$ and $b_{j}^{l}$. For example, if $z_{j}^{l}\geq 0$, then $z_{j}^{l+}=z_{j}^{l}$ and $z_{j}^{l-}=0$. By contrast, if $z_{j}^{l}<0$, then $z_{j}^{l+}=0$ and $z_{j}^{l-}=z_{j}^{l}$ *α* and *β* are coefficients partitioning unity and weighting the positive and negative parts.

In our relevance analysis we included only the training samples of training folds where *R* ^2^ ≥ 0.7 was reached for the validation sample to guarantee a high model accuracy and, thus, a high reliability of the relevance analysis itself. Our machine learning architecture consists of a standard CANN block and an additional deep neural network block for the computation of the Prony parameters. We performed our relevance analysis separately for both blocks. In both cases, we quantified the relevance of the 11 ELISA values. For the entire relevance analysis we used *α* = 2 and *β* = −1 in (14).

## 3 Results

### 3.1 Regional Microstructural Components Quantified Through ELISA

**FIGURE 5:**
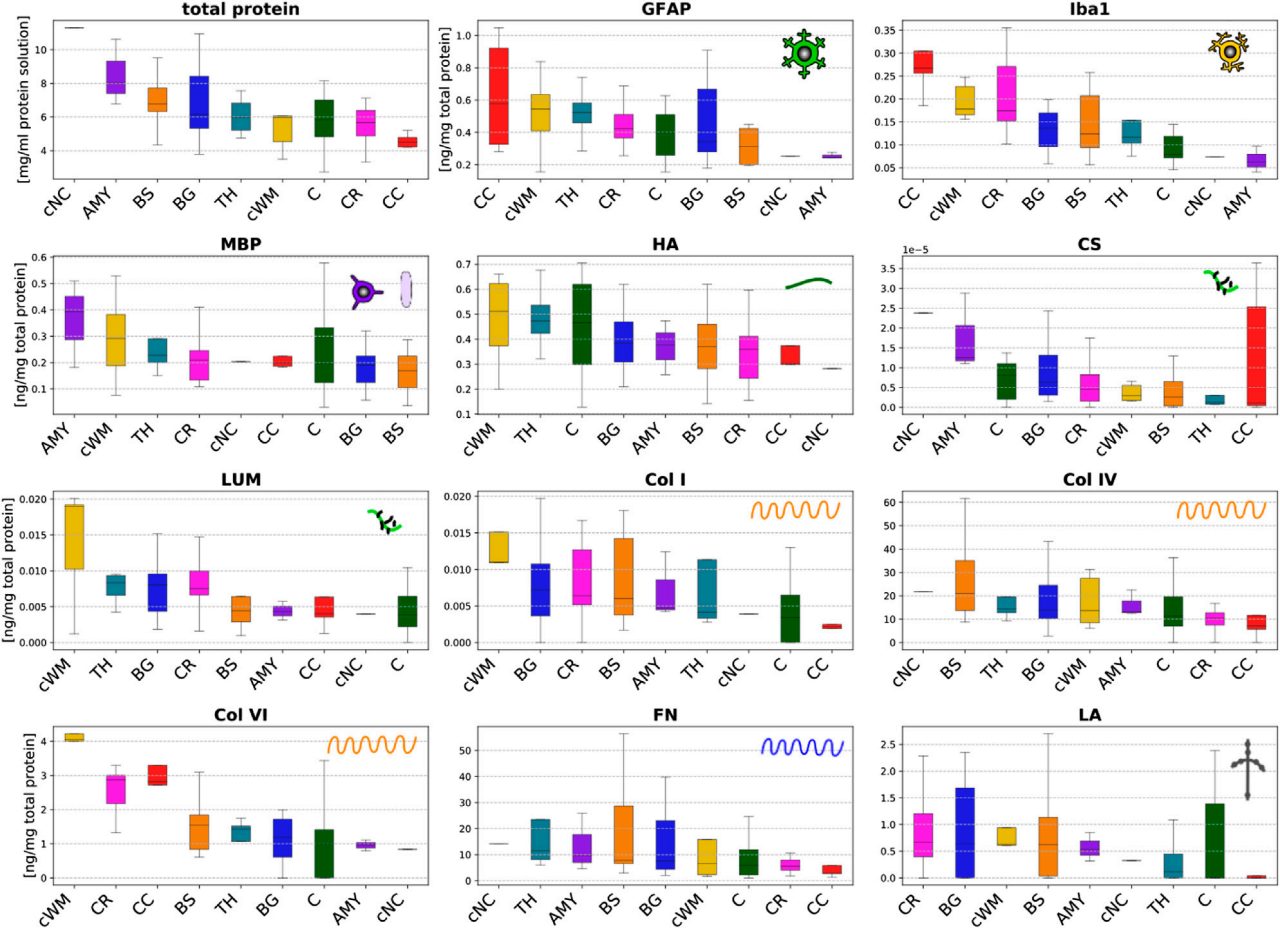
Total protein content (upper left) and protein mass fractions of 11 species (glial fibrillary acidic protein (GFAP), microglia- and macrophage-specific protein Iba1 (Iba1), myelin basic protein (MBP), hyaluronic acid (HA), chondroitin sulfate (CS), lumican (LUM), collagen I (Col I), collagen IV (Col IV), collagen VI (Col VI), fibronectin (FN), and laminin (LA)) evaluated by ELISAs in 9 different brain regions (cortex (C), thalamus (TH), basal ganglia (BG), amygdala (AMY), cerebral white matter (CR), corpus callosum (CC), brainstem (BS), cerebellar white matter (cWM), and deep cerebellar nuclei (cNC)).

Figure 5 summarizes the total protein content (per millimeter solution) and the results of the ELISAs reported in nanogram per microgram total protein for the different brain regions specified in Figure 1A. The total protein content quantifies the amount of proteins per milliliter solution. It ranges from 1 to 11.5 mg/ml solution. We note that these absolute values do not necessarily refer to how much proteins are present in different brain regions as they represent the protein content per milliliter solution but not the protein content per milligram tissue.

The ELISA results vary significantly for the different microstructural components introduced in Figure 2, and range from extremely small values on the order of 10^–5^ ng/mg total protein for chondroitin sulfate to values of up to 60 ng/mg total protein for collagen IV and fibronectin. Interestingly, the content of cellular proteins (GFAP, MBP, Iba1) is rather small compared to specific extracellular proteins (FN, Col IV).

The glial fibrillary acidic protein (GFAP) values quantifying the amount of the hallmark intermediate filament protein in astrocytes lie in the range of 0.1–1.1 ng/mg total protein. The GFAP concentration is highest in the corpus callosum, thalamus and cerebellar white matter, while it is lowest in the amygdala and deep cerebellar nuclei. The amount of GFAP in the brainstem is relatively low compared to all other white matter regions. The values for the microglia- and macrophage-specific protein Iba1 range from 0.04 to 0.35 ng/mg total protein. The amount of Iba1 is generally higher in white matter than in gray matter regions. The brainstem shows a lower content than other white matter regions, while the corpus callosum has the highest content of Iba1. The myelin basic protein (MBP) concentration lies in the range of 0.02–0.54 ng/mg total protein. It is highest in the amygdala and lowest in the brainstem. Most regions, including both gray and white matter, show a value of approximately 0.2 ng/mg total protein.

The hyaluronic acid (HA) content ranges from 0.13 to 0.71 ng/mg total protein. In the cerebrum, gray matter regions (TH, C, BG, AMY) have a higher amount of HA than white matter regions (BS, CR, CC). In the cerebellum, we observe the opposite trend with a higher HA value for the cerebellar white matter than the deep cerebellar nuclei. The amount of the proteoglycan chondroitin sulfate is at least two orders of magnitude lower than for all other proteins. Despite the exception of the thalamus with a relatively low value, gray matter regions have a higher content of chondroitin sulfate than white matter regions. The concentration of the proteoglycan lumican ranging from 0.002 to 0.032 ng/mg total protein is higher than for chondroitin sulfate but still low. Collagen I was the only fibrillar collagen we analyzed in the current work. Its content varies between 0 and 0.015 ng/mg total protein with highest values in the cerebellar white matter and lowest in the corpus callosum. Our results show that the most abundant collagen type in brain tissue is the non-fibrillar collagen IV with concentrations ranging from 0 to 62 ng/mg total protein. The collagen IV concentrations are higher in deep gray matter, the brainstem and the cerebellum than in the cortex, corona radiata, and corpus callosum. Collagen VI, another non-fibrillar collagen type, shows concentrations ranging from 0 to 4.3 ng/mg total protein. The collagen VI content is consistently higher in white than in gray matter regions. The fibronectin content ranges from 1 to 58 ng/mg total protein, similar to collagen IV. It also shows a similar regional distribution as collagen IV with lowest values for the cortex, corona radiata, and corpus callosum. The concentration of laminin lies between 0 and 2.7 ng/mg total protein.

### 3.2 Performance of Viscoelastic CANNs

**FIGURE 6:**
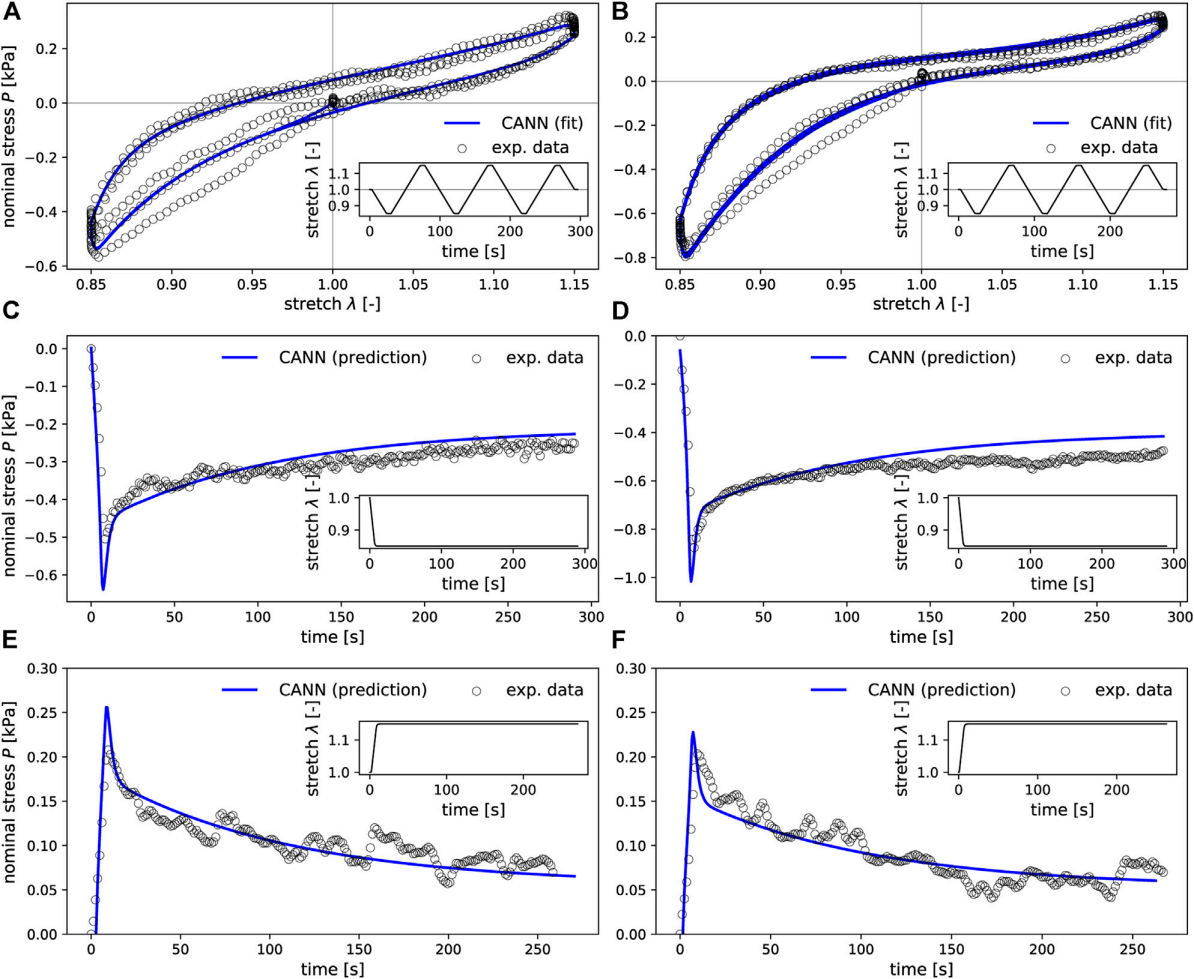
CANNs extended by the ability to account for viscoleastic effects and can learn to resemble the stress-strain curves provided as training data **(A,B)** and to predict the stress response also for loading scenarios not included in the training data such as the relaxation experiments shown in **(C–F)**. The first column **(A,C,E)** corresponds to mechanical data of one representative sample from the basal ganglia, while the second column **(B,D,F)** corresponds to a sample from the cerebellar white matter brain region.

**FIGURE 7:**
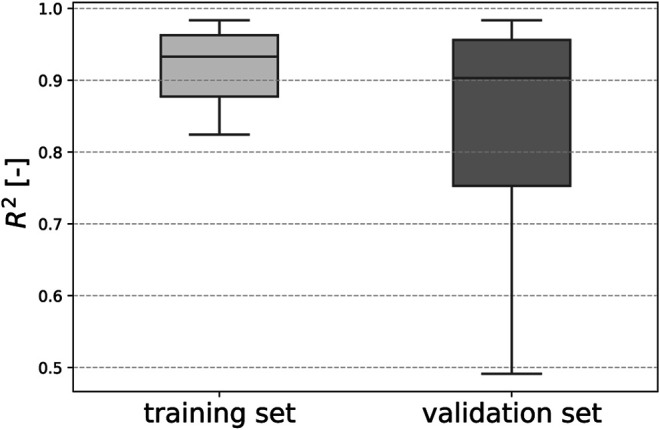
Our trained viscoelastic CANNs could reproduce the stress-strain curves in the training data with a median coefficient of determination *R* ^2^=0.94 and predict such curves for the validation samples with a median *R* ^2^=0.90 in a LOO-CV scheme.

In the training process, the neural networks employed in our study learned to resemble the stress-stretch curves provided as training data. Representative examples are depicted in Figures 6A, B. Moreover, the trained networks were able to predict the stress response of relaxation experiments not included in the training data, as shown in Figures 6C–F. Once trained for each fold in the LOO-CV scheme, the neural networks could reproduce the stress-stretch curves of the training data with a median coefficient of determination *R* ^2^ = 0.94 (standard deviation 0.24) and predict such curves for the validation samples with *R* ^2^ = 0.90 (standard deviation of ±0.51), see Figure 7.

### 3.3 Relevance Analysis Revealing the Link Between Mechanics and Microstructure

**FIGURE 8:**
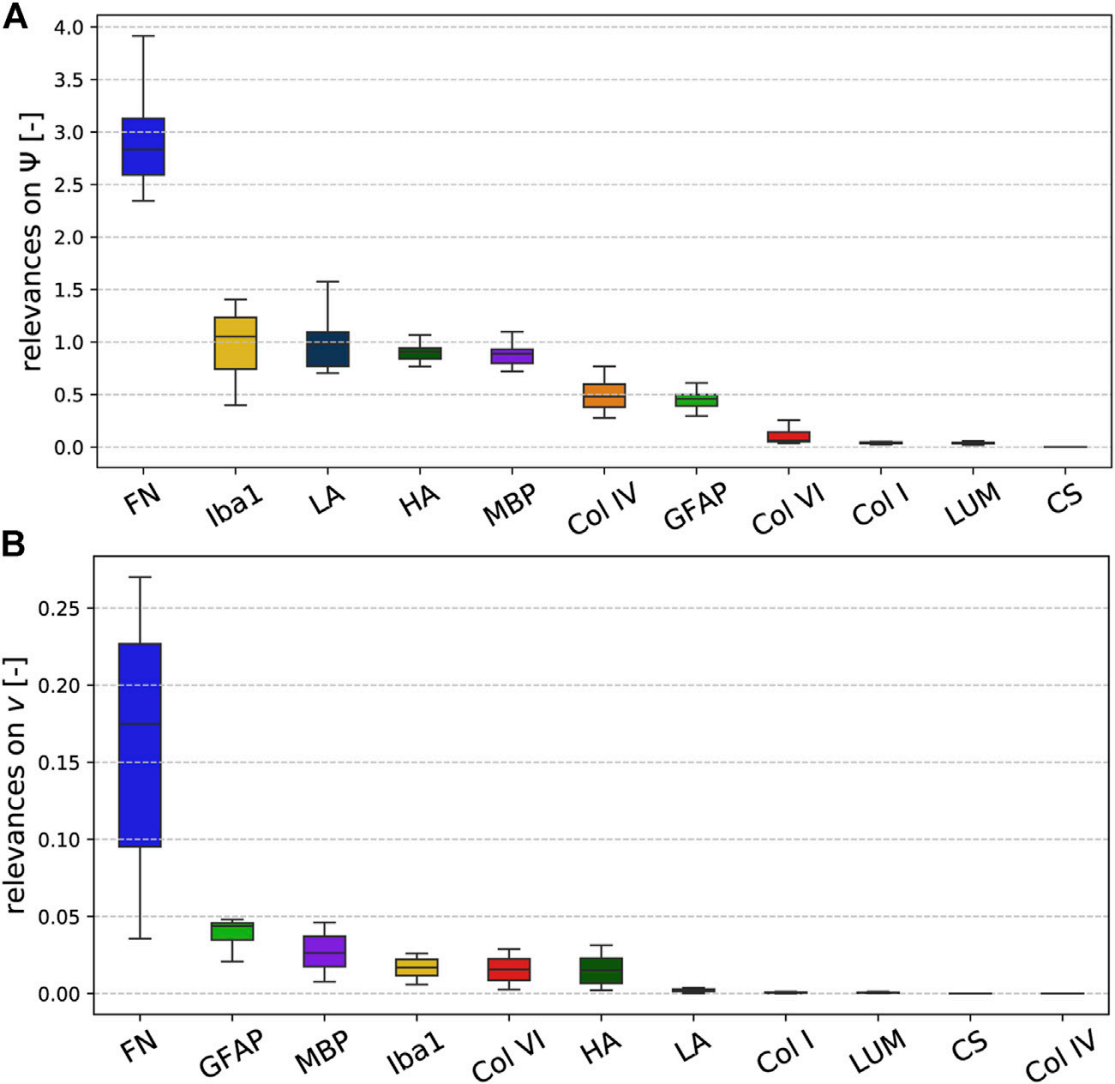
Relevance of the ELISA values for the **(A)** quasi-elastic stress response on very short time scales (governed by Ψ) and **(B)** viscoelastic effects (governed by the Prony series parameters collected in the set *v*).

Figure 8 illustrates the relevance (quantified through the backward pass in the viscoelastic CANN) of different microstructural components (quantified through ELISAs) for the quasi-elastic (Figure 8A) and viscoelastic (Figure 8B) contributions of the complex mechanical response of brain tissue. Fibronectin has the highest relevance for both the quasi-elastic response and viscoelastic effects. Concerning the quasi-elastic response, fibronectin is–with a certain distance–followed by Iba1 associated with microglia, the extracellular matrix proteins laminin and hyaluronic acid, as well as MBP associated with myelination of nerve fibers. Our results further suggest that collagen IV and GFAP slightly affect the quasi-elastic tissue response, while the influence of collagen VI, collagen I, lumican, and chondroitin sulfate seems to be negligible.

Concerning viscoelastic effects, interestingly all cellular components, quantified through GFAP (astrocytes), MBP (myelin, oligodendrocytes), and Iba1 (microglia), have the highest relevance after fibronectin. In addition, the extracellular matrix components collagen VI and hyaluronic acid seem to affect the viscoelastic behavior of brain tissue. We note that we find the lowest relevance for collagen IV, which is actually the protein with the highest amount per total protein, as illustrated in Figure 5. But, it appears to be irrelevant for the viscoelastic response of the tissue.

#### 3.3.1 Regional Trends for the Quasi-Elastic Stress Response

**FIGURE 9:**
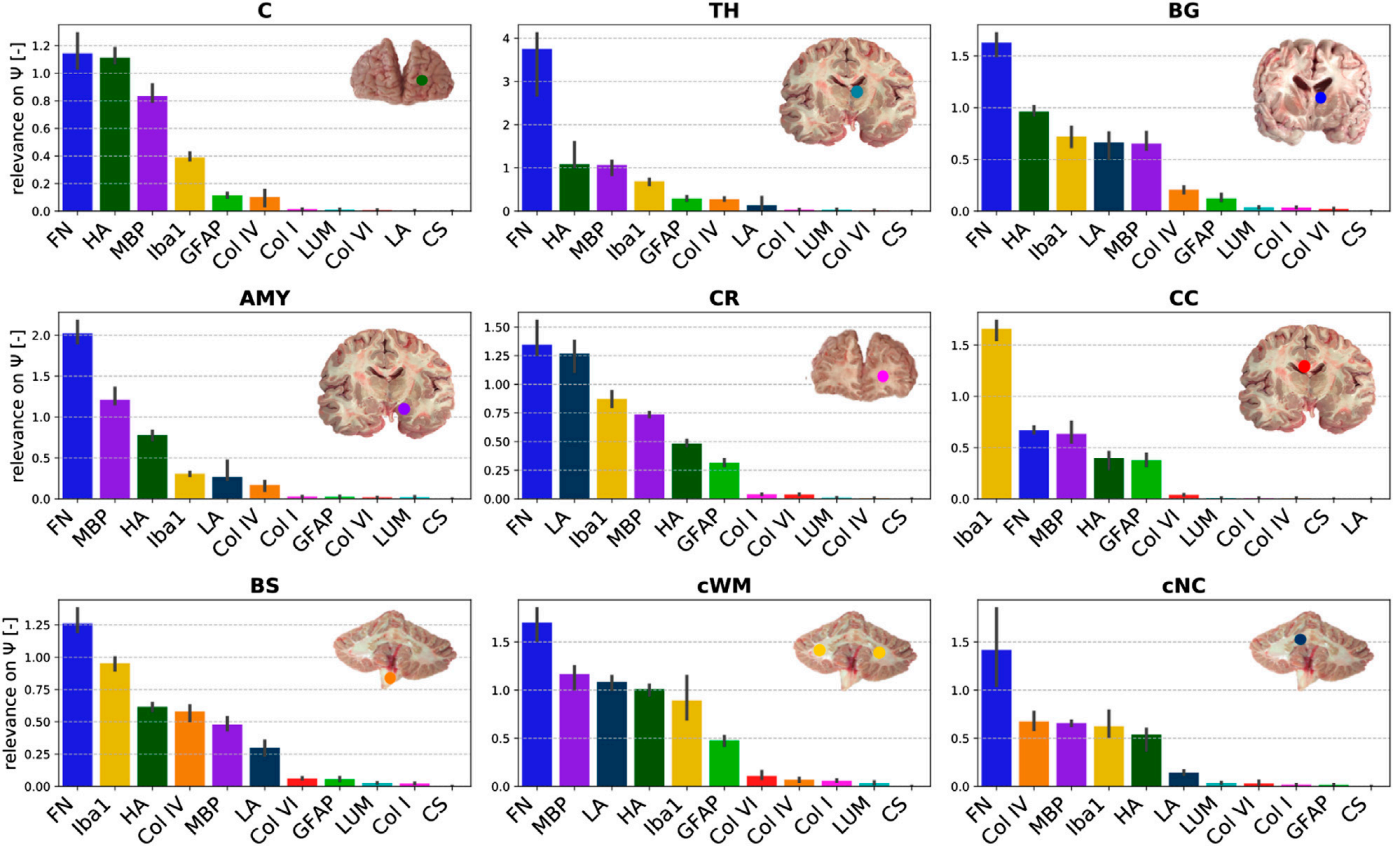
Relevance of the ELISA (see Table 3) values for quasi-elastic stress response (determined by Ψ) different brain regions (see Table 2).

Figure 9 displays the relevance of the different ELISA values for the quasi-elastic stress response in each brain region. The cortex and thalamus show a similar sequence. In the basal ganglia, laminin has a higher relevance than in all other gray matter regions, but the general trends are the same. In general, the relevance of Iba1 is higher for white matter than for gray matter regions. Furthermore, in all white matter regions (CR, CC, cWM) with the exception of the brainstem, GFAP shows a higher relevance than in gray matter regions. We observe that laminin has a relatively high relevance of approximately 1.2 in the corona radiata and cerebellar white matter. The relevance of hyaluronic acid lies on the order of 1 for the cerebral gray matter regions and cerebellar white matter, and around 0.5 for cerebral white matter regions and deep cerebellar nuclei. Interestingly, the only region, where fibronectin does not have the highest relevance, is the corpus callosum; here, Iba1 seems to control the quasi-elastic tissue response.

#### 3.3.2 Regional Trends for Viscoelastic Effects

**FIGURE 10:**
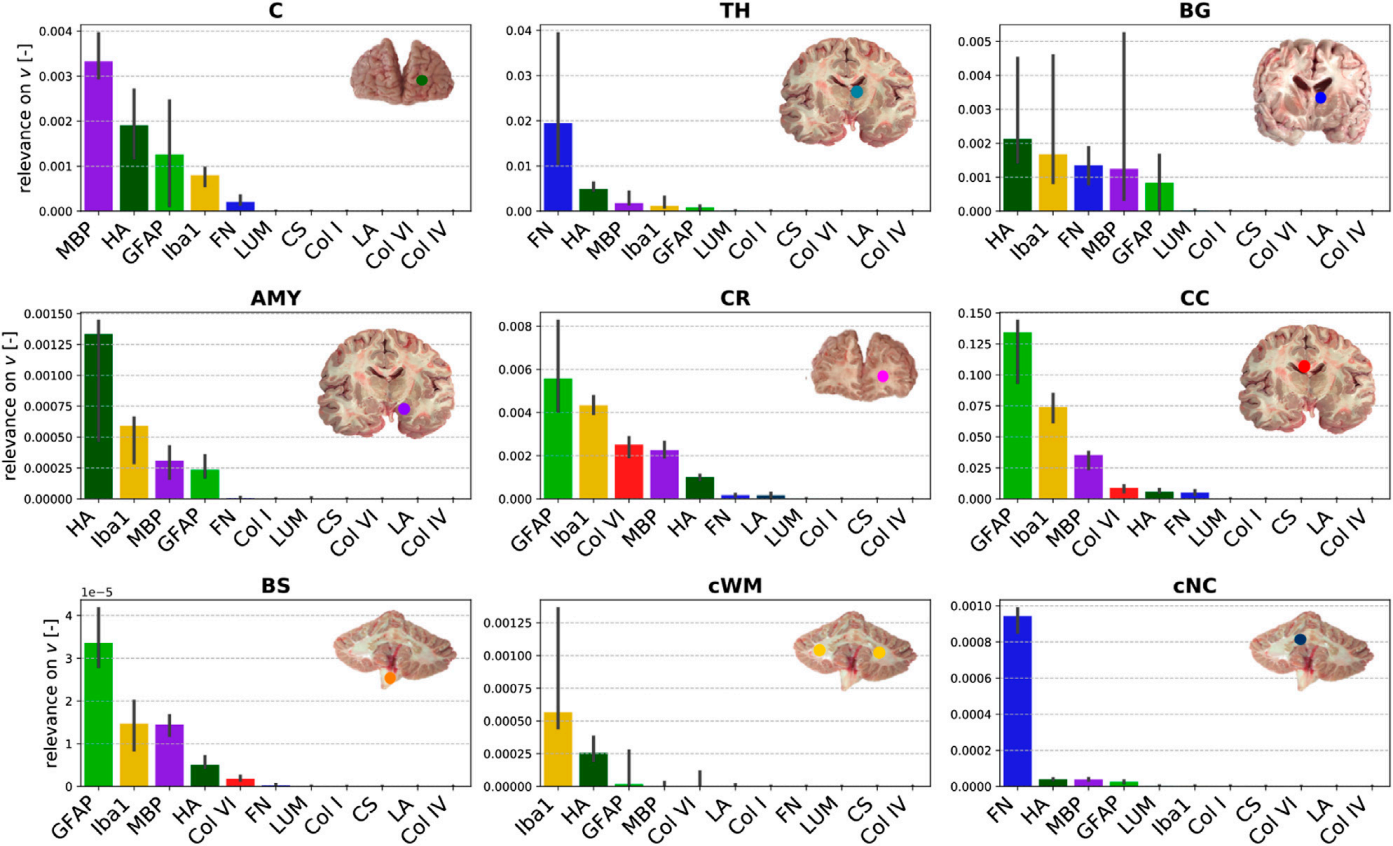
Relevance of the ELISA (see Table 3) values for the Prony series parameters in different brain regions (see Table 2).

Figure 10 displays the relevance of the different ELISA values for the viscoelastic behavior in each brain region. The regional trends are more diverse than for the quasi-elastic stress response in Figure 9. In different orders, MBP, hyaluronic acid, GFAP, Iba1, and fibronectin are most relevant for viscoelastic effects in gray matter regions. In cerebral white matter regions (CR, CC, BS), especially GFAP and Iba1 appear to play an important role. In addition, MBP, hyaluronic acid, and collagen VI show a certain relevance.

## 4 Discussion

In this study, we have combined mechanical large-strain compression and tension experiments (cyclic loading and stress relaxation) with microstructural investigations using enzyme-linked immunosorbent assays (ELISA) and an extended type of constitutive artificial neural network (CANN) that can account for viscoelastic effects to identify the link between the microstructural composition and complex mechanical response of human brain tissue.

### 4.1 Insights Into the Regional Microstructural Composition of Brain Tissue

To quantify the tissue composition in different regions of the human brain (see Figure 1), we have used ELISAs for selected cellular (GFAP - astrocytes, Iba1 - microglia, MBP - oligodendrocytes/myelin sheaths) and extracellular (hyaluronic acid, chondroitin sulfate, lumican, collagen I/IV/VI, fibronectin, laminin) proteins. The amount of cellular proteins was relatively low compared to certain extracellular components, which can be attributed to the fact that the investigated proteins only represent part of the cell. For instance, the myelin basic protein (MBP) represents 25–30% of all myelin proteins (Deber and Reynolds, 1991), and quantifies only part of the oligodendrocytes and myelin sheaths. We found MBP to be present in both gray and white matter regions. Interestingly, the MBP concentration was lowest in the brainstem. The microglia- and macrophage-specific protein Iba1 was more abundant in white matter than gray matter regions. This agrees well with a previous study reporting slightly higher densities of microglia in white matter tissue of different mammals (Dos Santos et al., 2020). Similar to Iba1, the astrocyte-specific protein GFAP was highest in the corpus callosum and the cerebellar white matter and lowest in the amygdala and cerebellar nuclei. The brainstem showed a significantly lower concentration of GFAP than all other white matter regions but was in the same range as the analyzed gray matter regions. This may be related to the fact that our brainstem samples included various small gray matter regions, such as the red nucleus and the substantia nigra in the midbrain, the pontine nuclei in the pons, and the medullary reticular formation and inferior olive in the medulla.

Overall, the most abundant proteins were fibronectin and collagen IV–both extracellular matrix components. Fibronectin is produced by endothelial cells, pericytes, and macrophages, and is predominant in perineural nets (Wang et al., 2011), as schematically illustrated in Figure 2. The high content of collagen IV agrees with findings in the literature reporting that collagen IV takes up about 50% of the basement membrane (Kim et al., 2018). Interestingly, the variation in the fibronectin and collagen IV content between different brain regions was relatively low, both having the lowest concentrations for tissue from the corpus callosum. Collagen VI was more abundant in white matter than gray matter regions with the highest content in the cerebellar white matter, closely followed by corona radiata and corpus callosum. For tissue from the cerebrum, hyaluronic acid (HA) showed the opposite trend with higher concentrations in gray matter than in white matter regions. This may be attributed to the fact that HA is an important component of perineuronal nets (see also Figure 2) that help regulate neuronal activity. In white matter, HA is more diffusely distributed around astrocytes and oligodendrocytes (Sherman et al., 2015).

The proteoglycan chondroitin sulfate appeared to be more abundant in the amygdala and the cerebellar nuclei than in all other brain regions–although its content was generally extremely low. In the amygdala, chondroitin sulfate is an important component of perineural nets (Pantazopoulos et al., 2008), and abnormalities in the chondroitin sulfate content are related to disorders like schizophrenia (Pantazopoulos et al., 2015). Similar to the proteoglycans, the concentration of fibrillar collagen I is particularly low in all brain regions, which is related to the ultrasoft mechanical response of brain tissue (Barnes et al., 2017).

### 4.2 Link Between Microstructural Composition and Macromechanical Properties

To quantify the mechanical properties of human brain tissue, we have introduced an extended type of CANN that incorporates substantial prior knowledge from materials theory and viscoelastic effects. It was able to learn the complex mechanical response of human brain tissue with a high accuracy over a large range of stress-stretch states. Moreover, it learned to predict the mechanical behavior of brain tissue from the 11 constituent concentrations measured through ELISAs. A layer-wise relevance propagation analysis allowed us to quantify the importance of the 11 individual constituent concentrations for the complex mechanical response.

The results of this analysis suggest that the content of fibronectin is by far the most relevant of the examined features for both the quasi-elastic stress response to loading and viscoelastic effects. This may be attributed to the fact that fibronectin forms fibrillar networks, which provide mechanical support. Interestingly, in a recent study on somite formation during embryogenesis, the fibronectin matrix was specifically perturbed to tune tissue mechanics (de Almeida et al., 2019). It has further been shown that the amount of fibronectin decreases in the aging brain (Syková et al., 1998; Wang et al., 2011), which may thus crucially contribute to the observed softening of brain tissue with age (Sack et al., 2009). Our results further indicate that the elastic tissue response is especially controlled by extracellular matrix proteins that are part of the basement membrane (Eriksdotter-Nilsson et al., 1986; Abhijit and Yao, 2019), i.e., fibronectin, laminin and collagen IV. Therefore, we suppose that the degree of vascularization plays an important role for brain stiffness, not least because we expect a higher stiffness for blood vessels compared to the brain parenchyma. It has been shown that both fibronectin and laminin are upregulated after traumatic brain injury (George and Geller, 2018). In addition, increased collagen IV and fibronectin signals were observed during ischemia (Michalski et al., 2020). This motivates the hypothesis that, in the future, altered mechanical properties could serve as a potential biomarker for such disorders. In addition to basement membrane proteins, hyaluronic acid (HA) appears to be relevant for the quasi-elastic tissue response. This agrees well with the general notion that HA plays the main structural role in the formation of the brain extracellular matrix (Bignami et al., 1993). Interestingly, HA appeared to be even more relevant for the elastic than for the viscoelastic response of brain tissue, which is surprising considering its hydrophilic nature.

The most relevant cellular protein for the quasi-elastic response is Iba1, which is specific to microglia and macrophages. Interestingly, microglia have been shown to preferably migrate towards stiffer regions (Bollmann et al., 2015). This could explain why the presence of microglia correlates with local mechanical tissue properties. The additional relevance of the cellular protein MBP also agrees well with previous findings showing that brain tissue stiffness correlates with myelin content (Weickenmeier et al., 2016; Weickenmeier et al. 2017b).

Viscoelastic effects seem to depend in particular on all cellular proteins, GFAP, MBP and Iba1, even though the absolute quantities of these proteins were rather low in all samples. This observation also agrees with our previous findings (Reiter et al., 2021), where we could show that the network of intercellular connections behaves viscoelastically. Interestingly, GFAP showed a higher relevance for the viscoelastic than for the quasi-elastic response of the tissue, which further supports the importance of the cellular network for brain viscoelasticity. In addition to fibronectin and cellular proteins, collagen VI showed a certain relevance, which we attribute to its ability to interact with fibrils and cells suggesting that it takes part in the viscoelastic network.

Despite the relatively high content of collagen IV in brain tissue, it is only moderately relevant for the quasi-elastic response and has the lowest relevance of all investigated proteins for the viscoelastic response. This demonstrates the effectiveness of the chosen approach using the backward pass through the viscoelastic CANN to evaluate the relevance of different microstructural components for complex brain tissue mechanics. The proteoglycans lumican and chondroitin sulfate and the fibrillar collagen I generally have only a very low influence on the mechanical properties of the tissue. They also exhibit the lowest concentrations.

### 4.3 Regional Differences in the Relevance of Constituents for Tissue Mechanics

When comparing the relation between composition and mechanical response of the tissue in different brain regions, we observe that relevances for the quasi-elastic tissue response are relatively insensitive towards the brain region. The only region that deviates from the highest relevance of fibronectin is the corpus callosum. Here, Iba1 seems to largely control the quasi-elastic tissue response. This might be related to the regional heterogeneity of microglia in the brain, where a different gene expression pattern was observed for the corpus callosum than for all other brain regions (Tan et al., 2020). Interestingly, GFAP is especially relevant in the corona radiata and corpus callosum–for the viscoelastic relaxation additionally in the brainstem–indicating that reactive astrocytes may significantly contribute to the mechanical response in these regions. Laminin was only relevant for the quasi-elastic response of the corona radiata, cerebellar white matter, basal ganglia, amygdala, and brainstem. In these regions, it was also most abundant. While fibronectin had a high relevance for the quasi-elastic response in all regions, it was also relevant for viscoelastic effects in gray matter regions. In general, we found that the regional trends were much more diverse for viscoelastic effects than for the quasi-elastic response. For instance, MBP was identified as most relevant in the cortex, fibronectin in the thalamus and cerebellar nuclei, HA in basal ganglia and amygdala, GFAP in cerebral white matter and the brainstem, and Iba1 in cerebellar white matter.

### 4.4 Implications for Microstructure-informed Constitutive Modeling

Independent of the brain region, fibronectin, HA, MBP, and Iba1 have a notable relevance for the quasi-elastic tissue response. Therefore, these constituents should be considered when developing refined microstructure-based material models for brain tissue in the future. We note, however, that ELISAs can only be performed *post mortem* or when tissue is surgically resected. Consequently, one may consider other techniques, potentially also *in vivo* imaging, to quantify the distribution of the constituents relevant for brain mechanics. Despite the relatively high content of collagen IV, our results indicate that its relevance for tissue mechanics is negligible. This could be attributed to the fact that collagen IV is a non-fibrillar collagen type. Also, fibrillar collagen I, which has previously been incorporated in material models for arteries (Gasser et al., 2006) or cartilage (Linka and Itskov, 2016), plays a negligible role for brain tissue mechanics as its concentration is very low.

With regard to viscoelastic effects, the most relevant constituents of the tissue seem to be Iba1, HA, MBP, and GFAP. As the relevance of the different proteins varied notably between different brain regions, it may be necessary to introduce region-specific constitutive models. According to our results, it might even be expedient to introduce different regional classifications for the quasi-elastic and viscoelastic contributions.

### 4.5 Limitations

As the samples for the ELISA analyses were extracted between 12 and 72 (brains 1 and 2) or 12 and 26 (brains 3–5) hours *post mortem*, some of the investigated proteins could already have degraded (Fountoulakis et al., 2001) and the mechanical response of the tissue could differ from the *in vivo* situation. When comparing the samples taken from the five human brains investigated here, which all reached our lab after different *post mortem* times, we did not detect noticeable differences in the protein content or mechanical response. Therefore, we anticipate that at least the comparison of the different brains and brain regions is reasonable. Here we focused on the relevance of different components on tissue mechanics and the comparison of different brain regions rather than only on determining the content of the individual proteins in the human brain.

### 4.6 Future Directions

In the future, we will further evaluate the predictive capabilities of the extended CANN framework. Potentially, it could be used to predict disease- or injury-related changes in tissue properties based on ELISA results performed on tissue extracted during a biopsy. In addition, it will be interesting to use a similar approach to predict the complex mechanical response of human brain tissue based on *in vivo* imaging data. In terms of the relation between microstructure and mechanics in the human brain, the next step is to not only consider the total amount of microstructral components, but also their morphology and three-dimensional arrangement. Concerning cellular components, it may be more reliable to quantify the number of nuclei instead of the concentration of GFAP and Iba1, which only represent part of the cell. In addition, we will consider the contribution of neurons and their connectivity.

## 5 Conclusion

In this study, we have followed a new paradigm by combining large-strain mechanical testing, enzyme-linked immunosorbent assays (ELISA), continuum mechanics theory and machine learning techniques to reveal the relation between human brain tissue composition and its mechanical properties. We introduced a viscoelastic constitutive artificial neural network model and were able to capture the mechanical response of the tissue during cyclic compression - tension experiments, and to predict the response during stress relaxation in compression and tension. By including the specimen-specific ELISA results into the network to model the mechanical response, and subsequently evaluating the backward pass through the viscoelastic CANN, we were able to reveal the relevance of the local tissue composition on the corresponding nonlinear and viscoelastic mechanical response. We have assessed the individual contribution of several cellular (GFAP, Iba1, MBP) and extracellular (hyaluronic acid, chondroitin sulfate, lumican, collagen I/IV/VI, fibronectin, laminin) proteins and evaluated region-dependent trends. Our results suggest that the extracellular matrix protein fibronectin has the highest overall relevance for both the elastic and viscous behavior of human brain tissue. While the quasi-elastic response seems to be largely controlled by extracellular matrix proteins from the basement membrane, cellular components have a higher importance for the viscoelastic effects. The tissue components relevant for the quasi-elastic response (fibronectin, hyaluronic acid, MBP, Iba1) are relatively insensitive towards the brain region. In contrast, regional trends for viscoelastic effects are more diverse. GFAP has a high relevance for white matter regions in the cerebrum and brainstem, and hyaluronic acid for most gray matter regions. Our results can have important implications for the development of microstructure-informed constitutive models to predict the regional behavior of brain tissue in finite element simulations. The latter promise to become a useful tool in assisting diagnosis and treatment of diseases or preventing injury. In addition, the relation between human brain tissue composition and mechanical properties facilitates the design of biomaterials for neural tissue engineering and 3D printing applications, where the investigated extracellular components could be valuable to enhance the biocompatibility and properties of matrix materials.

## Data Availability

The ELISA results and mechanical data presented in the study are included in the article/Supplementary Material, further inquiries can be directed to the corresponding author.
