# Supplementary material for: Unraveling the Local Relation Between Tissue Composition and Human Brain Mechanics Through Machine Learning
Source: Front Bioeng Biotechnol. 2021 Aug 17;9:704738. doi: 10.3389/fbioe.2021.704738 (PMC8415910; doi:10.3389/fbioe.2021.704738)
Supplement: Supplementary file 3 [file DataSheet1.pdf]

Supplementary Material

1 MODEL TRAINING AND VALIDATION

Model training and validation are closely related and essential in the development of all deep learning methods, since they reveal the descriptive and predictive capabilities of deep learning architectures based on the available data set.

1.1 Model parameter optimization

Deep learning model parameters (i.e. model weights) are typically determined iteratively in a model weight update scheme (model training), powered by gradient-decent optimization methods, to fit a given reference solution (supervised learning). In the present study, Adam optimization (Kinga and Adam, 2015) was adopted in the overall training process and all model weights of both deep learning blocks were obtained by minimizing the mean-squared-error (MSE) loss

$$\mathrm{MSE} = \sum_i |\mathrm{P}_{11}^i - \mathrm{P}_{\mathrm{exp}}^i|^2 \tag{S1}$$

with i looping through all tissue stretches included in the training data, P_{zz}^i is the stress component in loading direction as computed by our viscoelastic CANN and $\mathrm{P}_{\mathrm{exp}}^i$ is the corresponding experimentally observed value. Training was performed with 250 data pairs in each iteration (also referred to as batch size), which was chosen corresponding to the amount of cyclic stress-stretch data points of a single tissue specimen.The learning rate was fixed at 0.001 during the training of different layers. Glorot weight initialization was applied (Glorot and Bengio, 2010) as it is generally accepted as the best-practice to initialize the learnable model weights. This complete framework was implemented using the open-source software library and machine intelligence platform Keras with TensorFlow backend (Chollet, 2015; Abadi et al., 2020).

1.2 Leave-one-out cross validation

Leave-one-out cross validation (LOO-CV) was used to train the model on the full range of available data. Here, the number of evaluation folds is equal to the number of samples in the data set ($n = 86$). Accordingly, each model was first trained based on $n - 1$ data sets, and then the trained model was applied to the single left-out sample (validation sample) to evaluate the model generalization performance. Each model instance was trained for 4000 epochs, and the epoch with the best validation set accuracy was chosen for evaluation purposes. Such an approach provides an overview of the model performance by training it on the largest amount of available data. In addition, hyperparameter tuning was performed on cyclic loading data of one representative tissue specimen followed by the actual training process.

1.3 Hyperparameter tuning

The hyperparameter tuning involved the network topology, the dropout rate, L2-regularization and the activation functions and was conducted by a Bayesian optimization with a underlying Gaussian process

model as a built-in feature within the keras library (Mockus, 1994; Chollet, 2015). In particular, Bayesian optimization is used to define a meta-model in order to construct a parameter search-space. Here, this meta-model (also called fitness function) is defined by a Gaussian Process surrogate to estimate the CANN model performance in dependency of the employed hyperparameters. During the initial phase, a sparse sampling of the whole search space is generated, while in the subsequent phase, the sampling strategy focuses on favorable parameter regions of the fitness function. Ultimately, this enables an effective numerical method to identify an optimal set of hyperparameters to increase the CANN model performance. The resulting model architecture for the CANN was three hidden layers with [32, 32, 48] computational units with hyperbolic tangent activation functions, elu activation function (Clevert et al., 2015) on the output and a dropout layer after the first hidden layer with a rate of 0.5. The visco-network comprises a single hidden layer with 12 computational units and a sigmoid activation function.

2 SUPPLEMENTARY DATA

We have included the ELISA results in Supplementary Table 1 and the mechanical data in Data Sheets 2, 3, and 4, which contain individual folders for each loading condition (cyclic compression-tension, stress relaxation in compression, stress relaxation in tension) with text files for each sample.

REFERENCES

[Dataset] Abadi, M., Agarwal, A., Barham, P., Brevdo, E., Chen, Z., Citro, C., et al. (2020). TensorFlow: Large-scale machine learning on heterogeneous systems. Software available from tensorflow.org

[Dataset] Chollet, F. (2015). Keras. https://github.com/fchollet/keras

Clevert, D.-A., Unterthiner, T., and Hochreiter, S. (2015). Fast and accurate deep network learning by exponential linear units (elus). *arXiv preprint arXiv:1511.07289*

Glorot, X. and Bengio, Y. (2010). Understanding the difficulty of training deep feedforward neural networks. In *Proceedings of the thirteenth international conference on artificial intelligence and statistics*. 249–256

Kinga, D. and Adam, J. B. (2015). A method for stochastic optimization. In *International Conference on Learning Representations (ICLR)*. vol. 5

Mockus, J. (1994). Application of bayesian approach to numerical methods of global and stochastic optimization. *Journal of Global Optimization* 4, 347–365
